# Digitalization: potentials and pitfalls from a public health perspective

**DOI:** 10.1093/eurpub/ckz169

**Published:** 2019-11-18

**Authors:** Natasha Azzopardi-Muscat, Walter Ricciardi, Anna Odone, Stefan Buttigieg, Dineke Zeegers Paget

**Affiliations:** 1 Department of Health Services Management, Faculty of Health Science University of Malta, Villetta, Malta; 2 European Public Health Association, Utrecht, The Netherlands; 3 Sezione di Igiene, Istituto di Sanità Pubblica, Università Cattolica del Sacro Cuore, Rome, Italy; 4 School of Medicine, University Vita-Salute San Raffaele, Milan, Italy; 5 Digital Health Malta, Villetta, Malta

The ehealth conference organized under the auspices of the Maltese Council Presidency of the European Union in May 2017 will remain memorable for the plea made by Zsusanna Jakab for all experts and stakeholders to work for a ‘Beautiful Marriage’ between the worlds of digital health and public health.^1^

This supplement may be viewed as an outcome of the public health’s community response to this plea. Digitalization has become a driving force for transformation in all spheres of life. Health and health systems are not excluded from the influence of these developments. Yet, the health sector can be considered to be somewhat of a laggard when compared to other sectors and digital technologies have not yet impacted on health in the same way that they have impacted on other industries.^2^ Indeed, we still talk about ‘ehealth’ and ‘digital health’ when other industries that function almost exclusively through digitally enabled platforms have abandoned this prefix years ago.

The first article in this series by McKee et al.^3^ reminds us that this second information revolution is well underway. Whilst the benefits associated with digitalization include the power of information sharing amongst disparate communities as well as improved surveillance and diagnostics, the impact of other aspects of digital technology such as wearable devices on human health, may have been largely oversold. On the other hand, McKee et al. highlight five factors that should pose a serious cause for concern by the public health community and which merit further research. These are discrimination; breaches of privacy; iatrogenesis; disinformation and misinformation or ‘fake news;’ and cyber-attacks.

The harmful impacts of digitalization can be avoided if we have effective and appropriate governance mechanisms that are able to align digital innovation with public health system goals. Ricciardi et al. emphasize the onus on governments to create the policy environment and incentives that steer the industry towards the development, adoption and use of technologies that contribute to health system goals going beyond the confines of health technology assessment in evaluating specific technologies to see whether they should be funded.^4^

Azzopardi-Muscat and Sorensen highlight the importance of considering equity in the impact assessment of technologies, as well as in the type of policy and regulatory environment that digital technologies operate within. They propose the health literacy approach as one of the possible avenues to ensure that digital technologies work to reduce rather that reproduce health inequalities.^5^ Brall et al. build argue that it is imperative for digital health providers and regulators, to ensure that digital health interventions are designed and set up in an ethical and fair way if we wish to ensure a sense of ‘justice’ in the application of digital technologies in the health sector.^6^

Pastorino et al. document several initiatives that are advancing knowledge on the role of using big data for health. They call for new approaches to be found for translating the big data into meaningful information that health care professionals can use to impact on health outcomes and highlight the need for European action on international technical standards embracing a paradigm for openness in data.^7^ This will of course also require that health professionals are trained to discover the uses of such data to make a difference to their patients.

The final paper of this supplement by Odone et al. maps the potential of digital technologies to improve public health research, policy and practice. They show the importance and relevance of digital health to the various domains of public health practice. They also link the strategic objectives of the European Public Health Association to digital health action in areas including advocacy, evidence-generation, agenda-setting, capacity and knowledge building, training and leadership innovation.^8^

Our analysis and review supports the statement by the WHO Director General that ‘Ultimately, digital technologies are not ends in themselves; they are vital tools to promote health, keep the world safe and serve the vulnerable’.^9^ The public health community has a duty to engage with innovation but equally to uphold that the ethics and values which characterize the underpinning philosophical principles of our discipline are at the forefront of our endeavors. Only in this way, can we truly seek to exploit the potential for digitalization to enhance health and well-being whilst striving to avoid the pitfalls along the way.

*Conflicts of interest*: None declared.
